# Enhancing inferential abilities in adolescence: new hope for students in poverty

**DOI:** 10.3389/fnhum.2014.00924

**Published:** 2014-12-09

**Authors:** Jacquelyn F. Gamino, Michael M. Motes, Russell Riddle, G. Reid Lyon, Jeffrey S. Spence, Sandra B. Chapman

**Affiliations:** Center for BrainHealth, The University of Texas at DallasDallas, TX, USA

**Keywords:** inferential abilities, cognitive training, poverty, adolescence, gist reasoning, higher-order cognition, middle school

## Abstract

The ability to extrapolate essential gist through the analysis and synthesis of information, prediction of potential outcomes, abstraction of ideas, and integration of relationships with world knowledge is critical for higher-order learning. The present study investigated the efficacy of cognitive training to elicit improvements in gist reasoning and fact recall ability in 556 public middle school students (grades seven and eight), vs. a sample of 357 middle school students who served as a comparison group, to determine if changes in gist reasoning and fact recall were demonstrated without cognitive training. The results showed that, in general, cognitive training increased gist reasoning and fact recall abilities in students from families in poverty as well as students from families living above poverty. However, the magnitude of gains in gist reasoning varied as a function of gender and grade level. Our primary findings were that seventh and eighth grade girls and eighth grade boys showed significant increases in gist reasoning after training regardless of socioeconomic status (SES). There were no significant increases in gist reasoning or fact recall ability for the 357 middle school students who served as a comparison group. We postulate that cognitive training in middle school is efficacious for improving gist reasoning ability and fact recall in students from all socioeconomic levels.

As students reach adolescence, they strive to cope with the increased demands of advanced and more complex curricula. Their ability to make sense of and abstract meanings from information encountered through inferential processing is foundational to academic achievement (Brown and Day, 1983; Bunge et al., 2005; Chapman et al., 2006; Bunge and Wright, 2007). Unfortunately, recent studies (Stern and Ahlgren, 2002; Gamino et al., 2010) indicate that many students in middle school focus primarily on learning circumscribed details presented in textbooks without showing an ability to understand issues at a conceptual, in-depth level. Whether the problem rests in the superficial coverage of vast amounts of information in class or how the students are learning, evidence is mounting that critical gist reasoning (i.e., the ability to derive synthesized meanings by combining facts and applying inferential reasoning) is failing to develop in early adolescence—an age where the brain is at a critical stage to acquire advanced reasoning skills (Alberts, 2012). An over-reliance on straightforward fact recall in recent academic performance is readily apparent when one examines the downward trajectory of standardized state tests and National Assessment of Education Progress (NAEP) reading scores from fourth to eighth grade levels (Aud et al., 2011). The NAEP, administered at the eighth grade level, assesses the ability to glean deep meaning from texts through analysis and synthesis of information, inference of abstract concepts, prediction of outcomes, and relating what is presented in text to one’s own background knowledge.

Gist reasoning allows one to “connect the dots” between separate pieces of information, facilitating construction of generalized meanings rather than processing facts in isolation (van Dijk, 1995; Reyna, 1996, 2012; Chapman et al., 2006; Ryena and Mills, 2014; Wolfe et al., 2014). The ability to use inference to abstract meaning from incoming information (gist reasoning) is a skill that applies to formal as well as informal learning activities, such as reading a school assignment, listening and taking notes from a lecture, writing a class report on a specific topic, watching a movie or television program, or listening to a friend’s joke. Abstracting generalized meanings from class readings, for example, may be a more important indicator of meaningful, in-depth and efficient learning than recalling the specific facts (Brown et al., 1983; Ryena and Mills, 2014; Wolfe et al., 2014). Indeed, remembering exact wording has been found to be independent of remembering the inferential meanings conveyed in texts (Reyna and Kiernan, 1994; Wolfe et al., 2014). Research suggests that learning through gist-based concepts rather than trying to absorb and recite verbatim details supports long-term retention and decision-making (Ryena and Mills, 2014; Wolfe et al., 2014), affirming Reyna’s (2012) seminal theory that asserts the importance of gist to efficient memory and learning processes.

Brainerd and Reyna (1996, 2004) proposed a distinction within developmental trajectories between gist-based and verbatim memory processing (Reyna, 2012). Gist-based processing provides representations of semantic and relational information, or information abstracted from details. Conversely, verbatim processing provides representations of the explicit facts or concrete details. Whereas both gist and verbatim memory capacities generally have been found to increase with development, gist-based processing follows a more protracted trajectory (e.g., Brainerd et al., 1998). Reyna (1996) reported that meaning is rarely stored in its concrete/explicit form, but rather is quickly synthesized to a more generalized gist-based meaning.

We posit that gist reasoning involves goal-directed, frontally-mediated cognitive comparison processes that serve to enhance learning (Chapman and Mudar, 2014). Research in cognitive neuroscience has identified adolescence as a pivotal developmental epoch and critical window for acquiring reasoning and critical thinking skills in terms of both cognitive expansion and brain remodeling (Giedd et al., 2006; Yurgelun-Todd, 2007). In particular, adolescence is a critical life stage when executive functions such as advanced reasoning skills should be developing and expanding, with continued sophistication and refinement in adulthood (Blakemore and Choudhury, 2006; Bunge and Wright, 2007; Yurgelun-Todd, 2007). In particular, refinement of the connectivity within the frontal lobes is postulated to be a primary component of increases in working memory and the ability to hold and manipulate information (Bunge and Wright, 2007). The underlying neural substrates that support reasoning are undergoing dramatic change during adolescence (Yurgelun-Todd, 2007). Longitudinal neuroimaging research reveals extensive brain development and remodeling, particularly in frontal lobe networks, throughout adolescence and into early adulthood (Gogtay et al., 2004; Yurgelun-Todd, 2007), by way of pruning and strengthening neural connections. The complex frontal neural connections support higher-order cognitive functions such as problem-solving, decision-making, reasoning, judgment, and planning, and are often referred to as “executive control functions” (Sowell et al., 1999; Bunge et al., 2005; Bunge and Wright, 2007; Yurgelun-Todd, 2007).

Although extant studies have determined that cognitive training of executive functions such as reasoning skills promotes brain plasticity in children and young adults (Mackey et al., 2011, 2012), to date, few studies have examined gist reasoning growth trajectories among adolescents in the middle-school grades and even fewer have examined gist reasoning growth as a function of well-defined training protocols (Gamino et al., 2010). Previous research has implicated that both fact recall and gist reasoning competencies can be improved with a systematic strategy-based cognitive training approach (Gamino et al., 2010). However, the extent to which improvements are mediated by economic factors is not clearly understood, particularly with respect to whether living within a particular income level facilitates or hinders the magnitude of improvement. Poverty exerts a detrimental influence indirectly on both behavioral/cognitive and neurobiological outcomes via a collection of interacting mechanisms to include among others, maternal sensitivity, home environment, parental education, parent-child conflict, nutrition, and parental stress (Bradley and Corwyn, 2002; Mayer, 2002; Gershoff et al., 2003; Farah, 2010; Raver et al., 2012; Luby et al., 2013). Unfortunately, low-income environments tend to be associated with poorer nutritional, physical, and psychological conditions (Brooks-Gunn and Duncan, 1997; Mani et al., 2013). More importantly, children from low-income families often have reduced exposure to stimulating adult interactions and opportunities that positively influence vocabulary development and academic readiness skills (Gou and Harris, 2000; Fletcher et al., 2007). Thus, socioeconomic status (SES) may affect developmental trajectories in maturation of brain regions thought to mediate reasoning abilities and attenuate the gains of cognitive training beyond what is explained by the effect of age and gender alone.

In addition to SES, gender differences in higher-order cognitive functions are of interest given that the development of the neural systems, previously shown to be influenced by gist reasoning in adults (Chapman et al., 2013), differs for males and females. Specifically, male and female differences in developmental rates of brain size suggest that students in middle school, roughly 11–15 years of age, are in a transitional period when the brain’s complex frontal networks involved in higher-order thinking begin to undergo maturational changes (Stuss and Knight, 2013). Females reach maximum frontal volume approximately 1 year before males (11 vs. 12.1 years of age) and maximum parietal volume approximately 1.6 years before males (10.2 vs. 11.8 years of age) (Giedd et al., 1999; Klingberg et al., 2002; Gogtay et al., 2004). These data suggest that females tend to reach a state of anatomical maturation within brain regions thought to mediate reasoning abilities (Bunge et al., 2005; Jung and Haier, 2007) before males. This notion is further supported by findings that girls outperform boys on fluid reasoning tasks (Wright et al., 2008), suggesting that girls might be more responsive to cognitive training at an earlier age than boys. Understanding how gender affects gist reasoning ability and cognitive training outcomes will advance our knowledge of cognitive development and inform educational practices directed at promoting reasoning performance.

The present study explored the effects of strategy-based cognitive training on gist reasoning ability and fact learning among seventh and eighth grade public middle school students who varied in poverty status and gender. We examined whether cognitive training would increase gist reasoning abilities in seventh and eighth grade students, above and beyond that of typical development, which was validated in a separate comparison group. Furthermore, we explored the effects of gender and SES on response to cognitive training. Based upon previous research (Gamino et al., 2010), we hypothesized that students who received strategy-based cognitive training would show a significant increase on measures of gist reasoning and fact recall; whereas those without training would not show gains over a longer passage of time. Additionally, we hypothesized that adolescents from non-poverty level backgrounds would show greater training gains; however, even those from impoverished homes would show significant gains pre- and post-testing. With regard to gender, we posited that girls would show higher baseline performance on gist reasoning; however, both boys and girls would show significant gains in response to cognitive training. The goal was to ascertain the potential of cognitive training to improve the ability to infer meaning and recall facts for students living in poverty. This research is important given the evidence that a large percentage of students in middle school neither arrive in the seventh grade nor leave the ninth grade having become proficient in inferential thinking abilities (Alspaugh, 1998; Stern and Ahlgren, 2002).

## Methods

### Participants

A pre-post, quasi-experimental design, with a designated comparison group, was used for the current study. Experimental study participants were from public middle school seventh and eighth grade classes throughout Dallas, Texas and the surrounding urban and suburban area, with a total of 13 middle schools participating. Various teachers in each school were provided with the opportunity to allow their students to participate in the study, based on school district and/or campus administration suggestions/directives. Thus, the teachers who agreed to allow the research team into their classrooms volunteered to give up their instructional time in order to provide the opportunity for their students to participate in the study. Each school district and each school guided the seventh and/or eighth grade class/teacher selection without our input. Thus, the chosen teacher/classes consisted of students with various abilities and degrees of motivation, or lack thereof. Cumulatively, 1,031 students were offered the opportunity to participate in the cognitive training program. Of this initial pool, 140 students declined, leaving 891 students and their parents who signed informed assent and consent in accordance with our Institutional Review Board at the University of Texas at Dallas. Consent forms and family history questionnaires (FHQs) were proffered in English and Spanish. Of the 891 students with consent, complete pre-and post-cognitive training data were obtained for 741 participants. This initial group of students contained a majority of eighth graders (*n* = 480; 65%) with gender evenly distributed (48% male vs. 52% female). The average age of the students was 12.95 years (range 12–14 years), and included 32% Caucasian, 43% Hispanic, 17% African-American, and 8% Asian or other race. Students were given a total of $15 in restaurant gift cards for participating in the study. The gift cards were awarded sporadically throughout the data collection and intervention timeframes.

Parents or guardians completed an FHQ, on which they reported information regarding household income, number of family members living in the household, ethnicity, language spoken at home, the student’s developmental history, and any diagnosed medical or learning differences, such as a diagnosed brain injury, diagnosed learning disability, neurodevelopmental disorder, ADD or ADHD, or placement in special education courses. On the FHQ, 133 parents reported their child had, singularly or in combination, sustained a brain injury (*n* = 9), been diagnosed with a learning disability (*n* = 57), neurodevelopmental disorder (*n* = 16), or ADHD (*n* = 79), or been placed in special education courses (*n* = 38). Filtering out these children and those who had missing information on the FHQ yielded a sample of 556 participants having typical neurological development and on whom data analyses and poverty indexes were conducted; 209 of these participants were seventh grade students and 347 were eighth grade students.

To determine poverty status, we used an income–to-needs metric, using the United States census information to identify the discrete annual poverty level by taking into account the respective number of family members living in the home. This dollar figure was then divided into the respective family’s household income. A student’s family was defined as living in poverty if [household income]/[needs] < 1.0. Students not falling into this category were considered as living above the poverty line. Table 1 contains a description of this sample.

**Table 1:** **Sample characteristics for experimental group**.

		*N* and percent within grade	
**Gender**		Grade 7	Grade 8
Male		91 (43.5%)	151 (43.5%)
Female		118 (56.5%)	196 (56.5%)
**Poverty Status**
Male	Yes	38 (18.2%)	36 (10.4%)
	No	53 (25.4%)	115 (33.1%)
Female	Yes	44 (21.1%)	62 (17.9%)
	No	74 (35.4%)	134 (38.6%)
**Ethnicity**
African-American		43 (20.4%)	59 (17.0%)
Caucasian		33 (16.7%)	105 (30.3%)
Hispanic		121 (56.9%)	146 (42.1%)
Other		12 (6%)	37 (10.7%)
**Poverty × Ethnicity**
Poverty	Hispanic	58 (70.7%)	72 (73.5%)
	Non-Hispanic	24 (29.3%)	26 (26.5%)

We also recruited a separate sample of 357 sixth, seventh, and eighth grade students (176 males; 181 females), from both rural and urban middle schools, to form a comparison group (see Table 2). The assessment was extended to sixth grade students as the objective was to ascertain spontaneous development of inferential gist reasoning and fact recall in the absence of cognitive training across the middle schools grades, and eighth grade students matriculated to high schools prior to administration of a second assessment. This group of students formed three cohorts; students who were assessed in sixth grade and then reassessed in seventh grade, students who were assessed in sixth grade and reassessed in eighth grade, and students who were assessed in seventh grade and reassessed in eighth grade. The time points for assessment ranged from 10 to 16 months. This group of students did not provide information about parent income, learning disabilities, grade retention, head injury, or other potential confounding factors. We obtained information regarding ethnicity and SES levels through the participating schools’ general data collection and reporting. Thus, the comparison group, is a sample of typical public middle school students from Texas that did not exclude students from the group based upon learning disability, head injury, or any other health factors.

**Table 2:** **Sample characteristics for comparison group**.

	*N* and percent within cohort		
**Gender**	6th–7th Grade	7th–8th Grade	6th–8th Grade
Male	71 (43.8%)	81 (55.5%)	24 (49.0%)
Female	91 (56.2%)	65 (44.5%)	25 (51.0%)
**Economically disadvantaged**			
Yes	65%	64%	41%
No	35%	36%	59%
**Ethnicity**			
African-American	27%	26%	2%
Caucasian	33%	36%	90%
Hispanic	37%	35%	5%
Other	3%	3%	3%

### Gist reasoning measures

The Scale of Advanced Reasoning^©^ (SOAR^©^; Chapman et al., 2006; Gamino et al., 2010) was administered during one 45-min class period preceding and approximately 2 weeks following the cognitive training program to evaluate gist reasoning and fact recall ability for the experimental group. The average length of time between test administrations for the comparison group was 330.2 days (*sd* = 69.6).

The SOAR assesses an individual’s ability to spontaneously abstract and convey deeper meaning from a lengthy text, similar to those encountered in the classroom. The SOAR is a pen and paper assessment that consists primarily of three summarization tasks to determine gist reasoning ability and secondarily, probe questions to determine recall of pertinent facts. The SOAR entails three texts of differing lengths, two narrative and one expository, that participants are required to summarize. The students’ summaries are scored for the number of abstracted deeper meanings that are produced within the summary. Production of abstracted deeper meanings during summarization reflects the ability to spontaneously utilize gist reasoning to understand and convey unstated underlying ideas derived from information (Chapman et al., 2006; Gamino et al., 2009, 2010). In previous unpublished pilot studies involving samples of children 8–14 years of age, gist reasoning scores from the SOAR showed a significant correlation to scores on the Similarities (*r* = 0.57, *p* < 0.001, *n* = 93) and Vocabulary (*r* = 0.60, *p* < 0.001, *n* = 48) subtests from the Weschler Abbreviated Scale of Intelligence (WASI; Wechsler, 1999). However, as the WASI requires individual testing, we did not utilize the WASI for this study.

Prior to taking the SOAR, students were given instructions regarding the qualities of a good summary. Specifically, students were instructed that a summary is a well-organized, shortened version of the original text that conveys the bigger ideas and important information that can be understood from the text, much like summaries in a movie review or on a DVD cover. Additionally, the students were instructed that their summaries should contain enough information so that readers could gain an understanding of the original text. An example of a high-level gist-based summary of a common fairy tale, “Little Red Riding Hood,” was presented.

During testing, each passage was projected onto a screen for students to read and/or follow along while a proctor read aloud. After each text was read, students were reminded that they were to write a summary that included the important bigger ideas from the text. Subsequently, the summaries were collected, and the students were given a form with eight questions (i.e., fact recall probes) regarding the factual information that could be gleaned from the texts, and instructed to give short but complete answers to the questions. This process was repeated for each of the three texts. The testing took place at the students’ respective schools, during the regular classroom periods.

Summaries were scored via independent raters who were blinded as to whether the summary was baseline, post-cognitive training, or time one or two for the comparison group. The raters scored each idea conveyed in the summaries according to the manualized SOAR scoring rubric, as either a zero or one, depending upon whether it exemplified an abstracted, inferred meaning that was not explicitly stated in the text. Statements that were directly stated in the text were rated zero since the measure assesses the ability to integrate ideas into more generalized statements than originally presented in the text. The fact recall score was based upon the correctness and completeness of answers to probe questions, which were scored zero, one, or two points. Two trained raters independently scored each summary and the responses to the probe questions using the SOAR checklist rubric. The raters conferred to reach a consensus on final gist reasoning and fact scores, achieving inter-rater reliability of 92% and 94%, respectively; disagreements between the raters were subsequently resolved. The highest possible total score for gist-based concepts summed across all three texts was 25. The highest possible total score for fact recall summed across the three texts was 48.

### Cognitive training program

The cognitive training program used in the present study was the Strategic Memory Advanced Reasoning Training (SMART^©^) program developed at the Center for BrainHealth (Chapman and Gamino, 2008; Gamino et al., 2010). The SMART program trains students to use specific cognitive processes that foster top-down thinking and the ability to abstract meaning from information (Gamino et al., 2010). The SMART program consists of hierarchical cognitive processes that are explained and practiced through group interactive exercises and pen and paper activities using student instructional manuals. The seven processes entail: (1) deliberate inhibition of extraneous information; (2) chunking and organizing relevant information; (3) inference; (4) paraphrasing; (5) synthesis of important details; (6) interpretation of take home messages; and (7) abstraction of deeper meanings and synthesis of the processes in order to elicit top-down processing.

The instructed processes emphasize the integration of world knowledge with incoming facts in order to capture overarching themes and facilitate higher-order thinking (Mayer, 1989; Chapman et al., 2004, 2006; Gamino et al., 2010). In the first half of the program, students are specifically taught metacognitive aspects of abstracting meaning from information. The core focus includes cognitive skills such as selective attention which trains students to filter out less important information, chunking important facts into generalized ideas through inferential and interpretive paraphrasing, followed by synthesis of world knowledge to promote depth of understanding (Kintsch and Van Dijk, 1978). After the processes are introduced and practiced, the program emphasis turns to utilizing the foundation provided by the acquired metacognitive awareness to instill top-down processing from the onset of an assignment. Thus, students’ preliminary focus is on the deeper meaning of information prior to deliberate processing of isolated details. The texts and materials used within the program to practice the cognitive processes are similar to content that is typically encountered in English literature, social studies, and science texts. The manualized program was administered to students by trained research associates during regular classroom periods consisting of ten 45-min classroom sessions over a one-month period.

## Analyses

To determine the effect of cognitive training on gist reasoning ability for the experimental group, we applied a standard general linear model (GLM) to baseline and post-training gist reasoning and fact recall assessing the effects of gender, grade level (seventh or eighth grade) and SES. SES was coded as a binary variable determined by a ratio of family income to household needs (number of household members). A score less than 1.0 was designated as “poverty status”.

We also examined post-training gist reasoning while statistically controlling for the influences of pre-training gist reasoning and fact recall ability for the experimental group. Moderate pairwise correlations were found among baseline gist reasoning, baseline fact recall, and post-training fact recall, thus we applied a principal component (PC) reduction to derive two orthogonal variables that comprised nearly 88% of the total variability (see Table 3). The two derived variables, factor scores for each individual, were included as covariates in the GLM in order to account for their independent influences on post-training gist reasoning means. All two-and three-factor interactions were included in the GLM. PC derivation was implemented in the R statistical computing language^1^ and the statistical model was implemented in SAS (Cary, NC).

**Table 3:** **PC loadings to derive adjustment variables in GLM**.

	**PC1 Loading**	**PC2 Loading**
**Baseline GIST**	0.459	−0.867
**Baseline FACT**	0.649	0.179
**Post-training FACT**	0.606	0.465

## Results

### Gist reasoning scores

**Figure 1:**
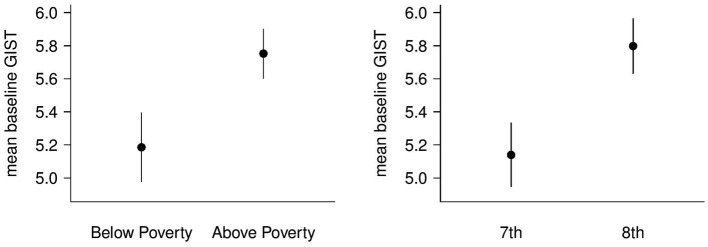
**Mean Baseline Gist Reasoning.** The figure on the left represents the mean baseline gist reasoning ability for students living below the poverty line and students living above the poverty line. The figure on the right represents the mean baseline gist reasoning ability for seventh and eight grade students.

At the initial assessment, seventh grade students in the experimental group demonstrated significantly higher gist reasoning ability than seventh grade controls, (*F*_(1,360)_ = 5.7, *p* = 0.02, *d* = 0.26 ). The analyses revealed significant SES and grade-level differences in mean gist reasoning scores prior to training for the experimental group. The students who were not living in poverty had significantly higher mean gist reasoning ability at baseline than students living in poverty (*F*_ (1,548)_ = 4.98, *p* = 0.026, *d* = [0.19, 0.23]); in addition, eighth grade students outperformed seventh grade students (*F*_(1,548)_ = 6.72, *p* = 0.010, *d* = [0.22, 0.27], see Figure 1). While no gender differences were found in the experimental group as a whole at baseline (*F*_(1,548)_ = 0.32, *p* = 0.57), eighth grade girls were found to have higher gist reasoning scores than eighth grade boys (*F*_(1,390)_ = 7.82, *p* = 0.005, *d* = 0.29). For the comparison group, no significant differences in gist reasoning ability at the initial assessment were found for gender or grade (*F*_(1,353)_ = 0.42, *p* = 0.52; *F*_(1,353)_ = 0.86, *p* = 0.36; respectively).

Mean gist reasoning scores significantly improved after cognitive training across the set of gender, SES, and grade-level combinations, (*F*_(2,548)_ = 58.28, *p* < 0.0001; *d* = 0.479), in spite of the differences found in baseline scores across SES, grade, and gender. The comparison group demonstrated no significant changes in gist reasoning ability from time one to time two, after controlling for grade level, gender, and number of days between testing (*F*_(1,349)_ = 0.73, *p* = 0.39).

#### Fact recall scores

**Figure 2:**
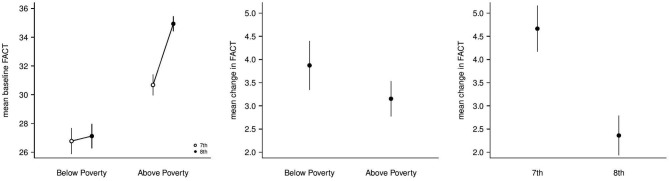
**Mean Fact Recall.** The figure on the left represents the mean baseline fact learning ability for seventh and eighth grade students living above and below poverty. The middle figure represents the mean change in fact learning ability for students living above and below poverty after cognitive training. The figure on the right represents the mean change in fact learning for seventh and eighth grade students.

There were no significant differences in fact recall ability between the experimental and the comparison group at the initial assessment. At baseline for the experimental group, fact recall scores among the students living above poverty were significantly higher than those who were living in poverty (*F*_(1,548)_ = 61.69, *p* < 0.0001; *d* = [0.70, 0.79]). Additionally, grade level contributed to baseline fact scores differentially within SES levels. As shown in Figure 2, at baseline eighth grade students living above the poverty line had significantly higher mean fact recall than seventh grade students living above the poverty line, but there was no difference in grade-level fact recall means for the students living in poverty (grade × SES interaction, *F*_(1,548)_ = 6.87, *p* = 0.009, *d* = [0.46, 0.53]). No gender differences were found for baseline fact recall ability, either at grade level or SES (*F*_(1,548)_ = 0.42, *p* = 0.51; and *F*_(1,548)_ = 0.17, *p* = 0.68; respectively) for the experimental group. For the comparisons, sixth grade females demonstrated significantly better fact recall than males at baseline (*F*_(1,209)_ = 4.14, *p* = 0.04), but no gender differences were found for seventh grade students. There were no significant differences between grades for the comparison group (*F*_(1,353)_ = 1.31, *p* = 0.25).

Following cognitive training, the students living in poverty had a comparable increase in mean fact recall scores relative to the higher income group. Seventh grade students demonstrated significantly more fact recall improvement after cognitive training than the eighth grade cohort (*F*_(1,548)_ = 12.83, *p* < 0.001; *d* = [0.31, 0.36], see Figure 2). The comparison group failed to show significant growth in fact recall between time one and time two after controlling for gender, grade level, and time elapsed between assessment (*F*_(1,349)_ = 0.26, *p* = 0.61).

### Effects of grade level, gender and SES on post-training gist reasoning

To account for pre-existing differences that might otherwise unfairly influence comparisons of post-training means, we adjusted the post-session gist reasoning scores by regressing them on the two PC-derived variables comprised of baseline gist reasoning score, baseline fact recall score, and post-training fact recall score (see Table 3 for the coefficients corresponding to each of these three variables). Effects of grade level, gender, and SES were assessed on the adjusted mean gist reasoning post-training scores.

**Figure 3:**
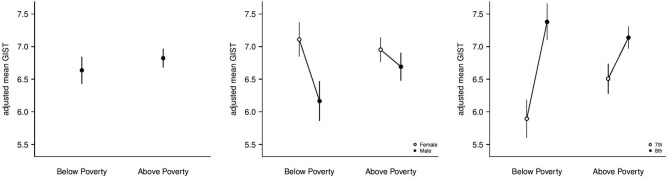
**Adjusted Mean Gist Reasoning Comparisons.** The figure on the left represents the adjusted mean gist reasoning ability after cognitive training for students living above and below poverty. The middle figure represents the adjusted mean gist reasoning abilities of boys and girls above and below poverty. The figure on the right represents the adjusted mean gist reasoning ability for seventh and eighth grade students living above and below poverty.

Following training, the students living in poverty demonstrated significant gains in gist reasoning scores that were statistically equivalent to the gains made by the students who lived above poverty (*F*_(1,546)_ = 0.54, *p* = 0.464, see Figure 3).

Although cognitive training significantly improved gist reasoning scores in both SES groups overall, gender and grade-level differences existed within each of the SES categories. We found that boys had a significantly lower mean post-training gist reasoning score than girls in the poverty level SES category (*F*_(1,546)_ = 6.07, *p* = 0.014; *d* = [0.33, 0.43]), whereas no significant gender difference was found for the group that did not live in poverty (*F*_(1,546)_ = 0.89, *p* = 0.345, see Figure 3, above). At the grade level, eighth grade students had significantly higher mean gist reasoning scores after training compared to seventh grade students both in poverty (*F*_(1,546)_ = 14.89, *p* < 0.001; *d* = [0.54, 0.65]) and above poverty (*F*_(1,546)_ = 5.02, *p* = 0.025; *d* = [0.22, 0.28], see Figure 3).

## Discussion

The present study is the first known research to specifically examine the efficacy of cognitive training to enhance gist reasoning and fact recall in a large and diverse group of seventh and eighth grade public middle school students as compared to typically developing students who received no specific training. All of our participants were recruited through their respective public middle schools and the assessments and training were performed during the regular school day. The participants came from a rich variety of socio-economic backgrounds, as well as school and home environments, providing ecological validity that exemplifies the melting pot of United States’ public school system. The diversity of schools and students who participated in our groups allowed us to examine the effects of poverty, gender, and grade level on the efficacy of cognitive training designed to enhance higher-order thinking skills.

Our study revealed that the gains attained in gist reasoning abilities after less than 10 h of cognitive training were superior to the improvements found in comparison students who did not receive training and who had a year or more to develop and improve gist reasoning through typical classroom teaching. One of the most important findings from this study was that middle school students living in poverty were able to harness cognitive plasticity by showing gains in gist reasoning similar to their more affluent peers. Our findings are similar to Mackey et al. (2011) who found children from a lower SES school demonstrated improved fluid reasoning after a two-month intervention. We postulate that intervening with low SES students with direct cognitive strategies to derive deeper meanings from complex information may attenuate the risk for delayed or stalled developmental trajectory of reasoning skills. Furthermore, we propose that investing time in cognitive training during regular school hours with middle school students regardless of SES could serve to enhance the development of higher-order thinking skills that exceeds the level attained through typical instruction. In particular, measures must be taken to put all students on an even playing field by providing opportunities to enhance academic outcome.

To put a finer point on it, what a child experiences living within a specific economic-level environment must be understood within a larger context that moderates those experiences, and provides opportunities to alleviate deficits in an effort to narrow the achievement gap. A wide variability exists in the presence and influence of mediating and moderating mechanisms within economic levels (Hackman and Farah, 2009). For example, increases in family income among poor families have greater positive impacts on children than increases among middle to high-income families (Raver et al., 2012). Poverty status has been associated with increased vulnerability of multiple brain systems (see Hackman and Farah, 2009 for review; Luby et al., 2013) particularly those involving frontal lobe development (Kishiyama et al., 2009), hindering the acquisition and use of executive control functions which include limitations in problem-solving, decision-making, reasoning, judgment, and planning (Sowell et al., 1999; Bunge et al., 2005). Extant research has found smaller volume in white and gray matter for school-aged children living in poverty (Luby et al., 2013), suggesting a hindrance to brain development that potentially renders children and adolescents limited in their ability to catch up with their more affluent peers. Modifying this previously held poor prognosis, our study found that middle school age students living below the poverty line made significant gains in gist reasoning. Similar to those of Mackey et al. (2011) our findings suggest that remediation of executive control functions is possible and worthwhile.

Our analysis indicated significant gains in gist reasoning ability for eighth grade boys and girls and seventh grade girls after cognitive training regardless of socioeconomic level. The eighth grade students showed significantly greater improvement in gist reasoning than the seventh grade students. The evidence herein suggests that seventh grade girls and eighth grade boys and girls are able to employ inferential processes after short-term, intensive cognitive training designed to improve higher order thinking skills. Specifically, seventh grade girls and eighth grade girls and boys demonstrated that they were able to abstract meaning when presented with new information from a wide variety of text-based information. These cognitive gains suggest that students demonstrated a developmental readiness to employ metacognitive strategies that enhanced understanding and the ability to infer global meanings from texts beyond the explicit facts, including students who were potentially subjected to deprived environments.

Although finding significant gains in gist reasoning across eighth grade students and seventh grade girls from above and below the poverty level was encouraging, we wanted to ascertain the extent to which pre-existing gist reasoning and fact recall abilities provided enhanced prospects for the efficacy of cognitive training. Pre-existing gist reasoning ability could be influenced by opportunities to attend better public schools, have greater access to books and learning opportunities, as well as exposure to positive/educational parental and adult interactions at home, with the resultant greater vocabulary exposure and acquisition. In other words, we sought to determine if the deleterious impact of growing up in poverty (Farah et al., 2006; Luby et al., 2013) would affect the efficacy of cognitive training for boys and girls in middle school. In order to determine if the gist reasoning gains found in students across socioeconomic levels were similar, we statistically co-varied baseline gist reasoning and fact recall ability. By leveling the baseline abilities of the students in our study, we could better determine if the students who lived in poverty were fundamentally disadvantaged and less apt to experience gains in higher-order cognitive skills with training.

Our analyses found that the students living in poverty showed significant increases in gist reasoning after training comparable to the gains made by their peers living above the poverty line. These results suggest that cognitive training may help reduce the academic achievement gap between socio-economic levels that plagues the United States. With direct training, eighth grade students and seventh grade girls were able to transfer cognitive processing skills learned during training to increase their ability to generate gist-based ideas from a text. In particular, the eighth grade girls who were living below the poverty level in our study showed significant gains in gist reasoning that allowed them to perform comparably to their higher SES peers. It is indeed encouraging to discover that children living in poverty will not necessarily succumb to academic stagnation, but may instead benefit from training to systematically build inferences to generate meanings that enhance understanding. A next step is to determine if cognitive training closes the academic achievement gap beyond the immediate assessment provided in this study, by collecting longitudinal data.

Whereas our findings indicate that the differences in gist reasoning performance between the eighth grade cohorts were not statistically significant, it should be noted that eighth grade boys living in poverty generally performed lower than any of the other eighth grade cohorts. It is possible that childhood poverty has a greater deleterious effect on brain development in boys than girls, such that cognitive training, while efficacious, does not yield the level of improvement of higher order processing at the middle school level in boys as it does for girls. It could be that an increase in the training duration would ameliorate the boys’ performance. On the other hand, intervening at an earlier age, especially for boys in poverty, may garner a larger increase in gist reasoning ability by eighth grade, as poor information processing habits might be curtailed with earlier intervention. Earlier intervention may increase boys’ confidence, as they could otherwise quietly acquiesce to deficient learning practices.

Failure to develop adequate gist reasoning skills during adolescence may have a profound and lasting effect on the individual in college and throughout adulthood (Willingham, 2009). While fact learning is important, the derivation of meaning by analyzing and synthesizing information, producing abstract concepts, predicting potential outcomes and inferring generalizable relationships and outcomes is absolutely essential for success in school and the workplace (Lehman and Nisbett, 1990; Nisbett, 1993). Theorists and educators recognize that these and other higher-order critical thinking skills typically undergo rapid expansion during adolescence and are refined in complexity and maturity throughout adulthood (Blakemore and Choudhury, 2006; Fischer et al., 2007). Thus, our study focused on middle school students, an age of extensive cognitive development (Bunge and Wright, 2007; Yurgelun-Todd, 2007) and expansion that is concomitant with an incumbent increase in academic demands.

Our findings suggest that seventh grade boys, regardless of SES, did not demonstrate significant gains in gist reasoning after cognitive training. We cautiously postulate that the slower trajectory of brain development in boys (Klingberg et al., 2002; Gogtay et al., 2004; Giedd et al., 2006) impinged transfer of the cognitive skills learned during post-assessment. To be sure, there are substantial individual differences in both age and physical, emotional, and social development among seventh graders that limits confidence in this potential explanation for the null findings. During the training sessions seventh grade boys demonstrated that they understood and could properly use the cognitive processes; however, without direct instruction to use their newly acquired skills, the seventh grade boys did not spontaneously apply what they had learned to the post-training assessment. Our findings are similar to those of Bjorklund et al. (1977), who found a lack of skill/strategy transfer in younger students but not older students after training. It may be that seventh grade boys require cognitive training of longer duration, or additional “booster” training sessions to obtain the benefits found in older children. Potentially, seventh grade boys may require a longer period of time to consolidate the processes acquired with cognitive training to render them useable, as all testing occurred within 2 weeks of the conclusion of the program. Alternatively, beginning cognitive training at an earlier age may prime the development of neural networks relevant to gist reasoning in seventh grade boys. More evidence is needed to determine if seventh grade boys would benefit from earlier intervention or from intervention of longer duration.

Our findings for an effect of gender and grade level on gist reasoning improvement differed from our findings for fact recall gains after cognitive training. We found significant gains in fact recall in both levels of SES, grades, and gender. Although the crux of the cognitive training program was not focused on basic recall, as discovered in previous studies (Gamino et al., 2010; Ryena and Mills, 2014; Wolfe et al., 2014), a focus on top-down processing of textual information positively influenced the ability to remember important details. Our finding supports extant research wherein interventions that utilize training protocols that consist of gist based concepts bolster fact recall (Ryena and Mills, 2014; Wolfe et al., 2014). Of particular interest to this study, the students living below the poverty line made gains in fact recall that were similar to the gains made by the students who did not live in poverty. As a whole, seventh grade students made greater gains in fact recall than eighth grade students. We postulate that when the ability to abstract meaning is purposefully developed, the synthesis of important details leads to greater depth of processing which enhances fact recall.

This study suggests that the deleterious effects of poverty on academic achievement (Farah et al., 2006) and brain volumes (Luby et al., 2013), may be reduced or ameliorated with cognitive training during the early adolescent years, a peak time for important brain development and connectivity refinement (Klingberg et al., 2002; Gogtay et al., 2004; Giedd et al., 2006; Yurgelun-Todd, 2007). Such findings of improved cognitive capacity in gist reasoning during adolescence indicate that the potential to harness cognitive and brain plasticity is preserved despite growing up in impoverished contexts (Mackey et al., 2011). Gist reasoning enhancement has been linked to improved brain function as measured by enhanced synchrony across networks and increased brain blood flow to complex frontally mediated networks in healthy adults (Chapman et al., 2013). With regard to the adolescent brain, Motes et al. (2014) identified enhanced frontally mediated inhibitory control on EEG measures in a subsample of adolescents who participated in the current study. Future studies should directly investigate the ability to strengthen cognitive capacity and underlying frontal networks during the critical developmental stage taking place in adolescence when the frontal networks and higher-order cognition are undergoing dramatic growth and reorganization. This goal is particularly relevant for youth from low-income backgrounds, to enhance their subsequent potential to succeed.

In an era wherein educational assessment frequently requires merely a regurgitation of facts, students are often more focused on memorizing huge quantities of information, rather than contemplating meaning and applying newly acquired understanding to novel situations (Stern and Ahlgren, 2002). This notion supports our finding that gist reasoning and fact recall abilities in our comparison group had not changed significantly a year or more after our initial assessment. It is likely that memorization does not enhance development of higher order processing, such as gist reasoning nor does memorization equate with understanding information at any depth other than surface level (Ryena and Mills, 2014; Wolfe et al., 2014). Public school curriculum in the United States generally provides breadth rather than depth of core subjects, leaving students with superficial information that has little relevancy in isolation. The evidence gathered herein reinforces the notion that improving gist reasoning in public middle school students, regardless of whether students live in poverty or not, may be attainable in fewer than 10 h of explicit instruction. Thus, the investment of time during the school day to promote direct instruction of higher-order thinking skills may prove invaluable to future academic outcomes that enhance college and career readiness. Unpublished longitudinal evidence gathered after a previous smaller study was published (Gamino et al., 2010) found students who received the cognitive training program graduated from high school on time at a higher rate than the school district average, with more than 70% of the students taking three or more Advanced Placement (AP) classes. Following the students in the present study longitudinally would provide evidence for the longer-term effects of cognitive training, and is a goal of the investigators.

### Limitations

The limitations of the present study include its cross-sectional design. Longitudinal data would provide evidence for the long-term efficacy of cognitive training for middle school students, especially seventh grade male students. In other words, longitudinal data may help to discern if training in seventh grade improves training outcomes for all eighth grade students, such that greater gains are demonstrated with continued cognitive training over two consecutive years. Additionally, collecting supplementary information about students’ involvement in early intervention programs, such as Head Start, would help us understand the factors that played a role in the efficacy of cognitive training. Future studies should include longitudinal data of academic markers such as grades and standardized test results to validate correlations of gist reasoning to academic success.

In addition to a lack of longitudinal data, we examined data of groups based on SES, gender, and grade-level, and thus our results are susceptible to design confounds commonly addressed through random assignment to groups. For example, factors associated with strategy acquisition or use, such as motivation or attention, may have influenced the observed differences between groups. Certainly, individual differences in such factors influence children’s acquisition and use of the strategies taught, and we attempted to statistically control for aspects of such differences with our covariate analysis. Additionally, for the observed group-level interaction effects, factors such as motivation or attention might have systematically varied with SES, gender, and grade-level (Gottfried et al., 1998) and might mediate differences in gains in gist reasoning. Indeed, SES, gender, and grade level are mere proxies for a host of biological and environmental variables (see, for example, Brooks-Gunn and Duncan, 1997; Bradley and Corwyn, 2002). However, gist reasoning gains on the SOAR, although affected by attention and motivation, show improvement in a student’s ability to synthesize the materials with world knowledge (i.e., although necessary, attention and motivation are not sufficient to lead to improved synthesis), and thus our data show synthesis gains following cognitive training even in the low SES groups. Future research should attempt to flesh out the degree to which such gains are mediated by changes in motivation and other factors.

Additionally, our comparison group was neither randomized nor participated in other training outside of their normal classroom activities, which are potential confounds. Likewise, our comparison group came from different schools than the schools that participated in the experimental group and we did not collect information about students’ diagnoses that would have enabled us to exclude participation as we did for the experimental group. None of the schools in which students participated in cognitive training enrolled their entire student body in the study, hence we were not given access to other students to recruit them to participate as controls or provide alternative training. The lack of control groups from the same schools as the experimental group was largely due to the disinclination by school administration to have a group of students within their schools that would not have equal access to a potential learning benefit. Therefore, we addressed the issue of a control group by extending an assessment-only option (comparison group status) to schools who did not receive cognitive training for their students immediately, but were assured that should our findings warrant and if funding became available, we would return to provide the program to their students. As such, the students who comprised the comparison group did not provide information regarding family income, learning disorders/differences, brain injury, or other potential confounds. Thus, the comparison group, while indicative of public middle school students, was not precisely matched to the intervention group with regard to those variables, and it is possible that this group lacked the motivation to perform at a higher level. In our previous randomized control study however (Gamino et al., 2010), wherein students in two different control groups were closely matched to the experimental group, we found no significant changes in gist reasoning ability of controls. Thus, we tentatively propose that our comparison group’s lack of growth in gist reasoning over time, may be indicative of many young adolescents in public middle schools wherein teaching students to pass high-stakes standardized tests takes precedence over depth of understanding.

**Figure 4:**
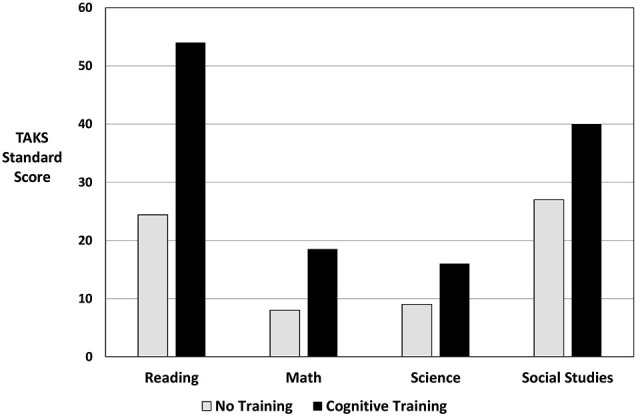
**Percent of students commended on the Texas Assessment of Knowledge and Skills from one low SES Dallas area campus;** the “No Training” group represents the eighth grade students from the same school who served as a quasi-control group.

The data presented herein represent the direct assessment of the ability to produce gist-based ideas and recall facts in seventh and eighth grade students who either received cognitive training or did not. Unfortunately, it reflects neither actual academic data nor the academic improvements one would hope to find after cognitive training. From a qualitative standpoint, many of the teachers in whose classrooms we conducted training reported increases in their students’ learning performance and standardized test scores. One eighth grade teacher from a low SES school provided data regarding the percentage of “commended” standardized state-mandated test scores from the Texas Assessment of Knowledge and Skills (TAKS) that were received by the students in her class who participated in cognitive training vs. the remainder of the campus. Commended scores represent the highest performance level a student can attain on the TAKS. Figure 4 compares the percentage of eighth grade students who did not participate in this study and received commended TAKS scores in various content areas compared to the percentage of students from the same low SES campus who received commended scores after participating in the experimental group. Thus, this graph does not represent the comparison group discussed herein but instead represents a cohort of students that is directly linked to a subset of our experimental group as they were all from the same middle school. The campus as a whole was not selected by the administration to participate in this research, thus the majority of the campus was compared only on standardized test results with the small group of students who were in the study. All eighth grade students from the school, the small experimental group, and the student body as a whole where included for this comparison, without excluding any of the students (including the experimental group) for various learning disabilities, head injury, or other factors. The graph indicates that the percentage of students in the cognitive training program who were commended exceeded the percentage of their peers across all content areas tested. While limited and with our acknowledgement that there are potential confounds when comparing these two groups of students, these academic markers of success are encouraging and suggest the necessity for continued study of the efficacy of cognitive training for improving academic performance and advancing career readiness.

In addition to the lack of academic data, our method for ascertaining the SES of our participants entailed using reported family income level and family size; however, additional information could have provided a more accurate portrayal of the students in our study. Poverty levels are frequently indexed through a determination of SES which, in turn, is typically conceptualized as a combination of parental education, income, and occupation (Sirin, 2005). Other studies have relied upon composite SES measures to include parental income, parental education, and parent occupation (Raver et al., 2011). However, there is wide variation in how SES is defined and measured in research and policy contexts (Bornstein and Bradley, 2003). Relying on a needs-to-income ratio may not have yielded a completely thorough appraisal of the poverty status of the students in our study; nevertheless we believe that our method provided a reasonably accurate assessment of our participants’ SES level.

## Conclusion

To be sure, the goal of education is to guide students to become strategic learners wherein they develop the skills to explore and comprehend topics in-depth. The goal-directed behavior to infer global meanings is non-negotiable if one is to be successful in school and the work place (Willingham, 2009). Indeed, memorizing facts does not equate to understanding and represents a formula for failed potential, particularly in children from poverty, who often have limited resources for improving their academic competencies.

Cognitive training in middle school, a time when brain development is at its peak (Giedd et al., 2006), may be the boost students from all income levels need to strengthen their confidence and invigorate their emerging cognitive abilities for the academic rigors that lie ahead in secondary school and beyond. Providing cognitive training at this age in particular, has the potential to take advantage of the brain’s plasticity to establish and strengthen the complex frontal networks. This study provides important evidence that, well beyond the early school years, students who have the misfortune of living in poverty can benefit from cognitive training as much as their more privileged peers. The evidence provided herein suggests that utilization of cognitive training within the public school system has the potential to reduce the academic achievement gap, which is underscored by socio-economic disparity.

From a practical perspective, the need for robust gist reasoning skills is readily apparent, for example, when one examines job announcements for entry-level management positions. Such positions routinely call for the ability to rapidly understand, utilize, and apply new knowledge in the conception and execution of tasks and to make flexible decisions on the basis of time constraints and limited resources. Our findings support previous reports that students demonstrate increases in the ability to understand and use information to flexibly problem solve with guided practice of higher-level cognitive processes (Gamino et al., 2010), perhaps increasing their employability in the future. Likewise the findings in this study support the theoretical position of Reyna (2012), that abstracting gist-based meaning is important for long-term retention of information and efficient learning.

This study may help inform middle school educators, public school administrators, and policy makers of the benefits of directly training top-down cognitive skills as a way to foster high-order thinking in middle school students. Training studies such as this can lead directly to an understanding of dosage and program duration effects to adjust instruction for various grade levels of students. Moreover, the results of studies such as this one have been found to be more influential in motivating educators’ attention to and use of research than studies which, at first blush, may not appear relevant to daily classroom practices (Lyon and Esterline, 2007). In essence, treatment studies such as this provide a powerful experimental context to obtain answers to this question: “For which children who vary in economic level are which treatment components and treatment dosages most beneficial and at what ages/grades, provided by which teachers, within which classrooms, within which schools” (Lyon, 1999). More studies are needed to supplant ineffective educational practices with evidence-based remedies.

## Conflict of interest statement

The authors declare that the research was conducted in the absence of any commercial or financial relationships that could be construed as a potential conflict of interest.

## Footnotes

^1^http://r-project.org
